# The Role of Mean Platelet Volume (MPV) in Thyroid Cancers: A Scoping Review

**DOI:** 10.3390/medicina62010100

**Published:** 2026-01-02

**Authors:** Andrei Alexandru Andoni, Florentina Severin, Alina Calin, Florin Mocanu, Ionut Andrei Roman, Octavian Dragos Palade, Roxana Grigorovici, Alexandru Grigorovici

**Affiliations:** 1 Surgery Department, Faculty of Medicine, “Grigore T. Popa” University of Medicine and Pharmacy, 700115 Iasi, Romania; andoni_andrei-alexandru@d.umfiasi.ro (A.A.A.);; 2 ENT Department, Faculty of Medicine, “Grigore T. Popa” University of Medicine and Pharmacy, 700115 Iasi, Romania; 3 Gastroenterology Department, Faculty of Medicine, “Grigore T. Popa” University of Medicine and Pharmacy, 700115 Iasi, Romania

**Keywords:** mean platelet volume, MPV, papillary thyroid carcinoma, thyroid cancer, biomarkers

## Abstract

*Background and Objectives*: Mean platelet volume (MPV) is a routinely available blood marker that measures platelet size and activation, and it has been evaluated as a potential marker for thyroid malignancies. Platelets participate in tumor genesis through angiogenesis, immune evasion, and metastasis, making them plausible adjuncts for cancer risk evaluation. The objective is to systematically evaluate the role of MPV in thyroid cancers, with the main focus on diagnostic accuracy, prognostic value, and limitations, focusing on papillary thyroid carcinoma (PTC). *Materials and Methods*: A systematic search of PubMed was conducted from January 2015 to September 2025. Only free full-text studies on human subjects were included. Eligible studies included case–control, cohort, or observational designs reporting MPV or platelet indices in thyroid cancer compared with benign nodules or healthy controls. Data on diagnostic performance, associations with tumor stage, lymph node involvement, and recurrence were extracted and synthesized narratively. No formal risk-of-bias or study quality assessment tool was applied. The literature search was restricted to studies with freely available full-text articles, which may have introduced access-based selection bias. *Results*: Eleven studies met the inclusion criteria. Most of them reported high MPV values in papillary thyroid carcinoma (PTC), with limited evidence regarding other thyroid cancer subtypes. High values of MPC were reported in the majority of studies in PTC compared to benign nodules or healthy controls. The diagnostic performance of MPV alone was poor, but integration with inflammatory ratios such as the neutrophil-to-lymphocyte ratio (NLR) or platelet-to-lymphocyte ratio (PLR), and with ultrasound systems (TI-RADS), improved accuracy. Regarding prognostic utility, some studies linked higher MPV with lymph node involvement or recurrence risk, while others did not find significant data. Thyroid function, autoimmune thyroid disease, and methodological variability in MPV measurement limited comparability across studies. *Conclusions*: MPV is a low-cost adjunct biomarker, especially when combined with other hematologic and imaging markers. However, MPV should not be used as a stand-alone diagnostic or prognostic tool. Larger, prospective studies are mandatory to clarify its clinical role.

## 1. Introduction

The incidence of thyroid carcinomas has rapidly increased in recent decades, being one of the most common type of endocrine malignancies, up to four times more common in women than men. Among the histopathological variants, the papillary thyroid carcinomas (PTCs) has had the most increased incidence [1]. Because diagnostic uncertainty persists in distinguishing malignant forms from benign or inflammatory thyroid disease, a growing interest in low-cost biomarkers has arisen to improve risk stratification in thyroid nodules. For diagnosis, fine-needle aspiration biopsy and ultrasonography remain the gold standard. In recent years, other parameters have been studied in order to differentiate between chronic inflammation and carcinogenesis [2]. Although many of these low-cost biomarkers have been explored, few of them have proven clinically useful.

A substantial number of studies conducted in recent years have now confirmed that chronic inflammation more often stimulates than inhibits tumor development [3]. Chronic inflammation plays a critical role in initiating, sustaining, and advancing tumor growth [4,5]. The possible mechanisms by which inflammation can contribute to carcinogenesis include induction of genomic instability, alterations in epigenetic events and subsequent inappropriate gene expression, enhanced proliferation of initiated cells, resistance to apoptosis, aggressive tumor neovascularization, invasion through the tumor-associated basement membrane, and metastasis [6,7]. The tumor itself can promote an inflammatory reaction via the secretion of proinflammatory factors or via the development of tumor necrosis [2,8,9]. The interaction between platelets (PLT) and cancer remains incompletely understood. However, accumulating evidence indicates a bidirectional relationship: malignant cells can drive platelet production and promote their activation and aggregation, while, in turn, platelets facilitate tumor growth, tissue invasion, and metastatic spread [10]. Regarding thyroid cancer specifically, the inflammatory tumor environment, alterations in thyroid hormone levels, and even autoimmune thyroid disease may affect platelet activation and volume, which provides a biological rationale for investigating platelet-related markers in thyroid malignancies. However, these mechanisms are shared across many malignancies.

Hematological parameters such as neutrophil and lymphocyte counts, the neutrophil-to-lymphocyte ratio (NLR), the lymphocyte-to-monocyte ratio (LMR), platelet count, and platelet indices (including platelet distribution width, plateletcrit, and mean platelet volume) reflect platelet morphology and activation. These parameters have been studied not only in cerebrovascular, thromboembolic, cardiovascular, and inflammatory diseases but also as potential inflammatory biomarkers in cancer patients [11]. However, their role in PTCs remains inconsistent, with studies reporting conflicting results. Despite repeated investigations, MPV has shown limited clinical significance. Beyond leukocyte-based indices, platelet-related markers have also been evaluated. Several studies have found significantly higher mean platelet volume (MPV) levels in PTC patients compared with healthy controls and patients with benign nodular goiter [12]. However, these differences are generally modes and vary across studies. MPV is always reported in complete blood counts and reflects platelet activation more directly than platelet count alone, being influenced by inflammatory, endocrine, or immune factors relevant to thyroid pathology.

MPV was reported to decrease significantly after thyroidectomy in PTC patients, a trend not observed in benign nodular goiter cases [13]. Some studies found no significant relationship between PTC and inflammatory hematological parameters, including, in particular, MPV [14]. The persistent but inconsistent associations reported for MPV in thyroid cancer suggest that it represents a biologically plausible yet clinically weak biomarker. So, this review aims to synthesize and evaluate the existing information available on MPV in thyroid cancer, with focus on their diagnostic and prognostic significance. Therefore, the main objectives of this review are to evaluate the prognostic value of MPV in thyroid cancer (regarding tumor stage, lymph node involvement, and recurrence), to establish the limitations in clinical applicability when thyroid function status is altered and autoimmune thyroid disease is present, and, finally, to determine whether MPV differs between malignant and benign thyroid nodules.

## 2. Materials and Methods

A comprehensive search was conducted in the PubMed database for articles published between January 2015 up to September 2025 to establish whether MPV values could have a diagnostic and prognostic value in thyroid cancer. Search terms included combinations of “mean platelet volume”, “MPV”, or “platelet indices” with “thyroid cancer”, “thyroid carcinoma”, or “papillary thyroid carcinoma”. No other databases (e.g., Embase, Web of Science, or Scopus) were searched. The search was restricted to PubMed because it provides a full coverage of the biomedical and clinical literature and ensures access to relevant studies. Only articles with free full-text availability were included to allow complete data extraction. This restriction may represent a source of selection bias and is acknowledged as a limitation of this review. The findings of this review should be interpreted as exploratory rather than definitive.

**Figure 1:**
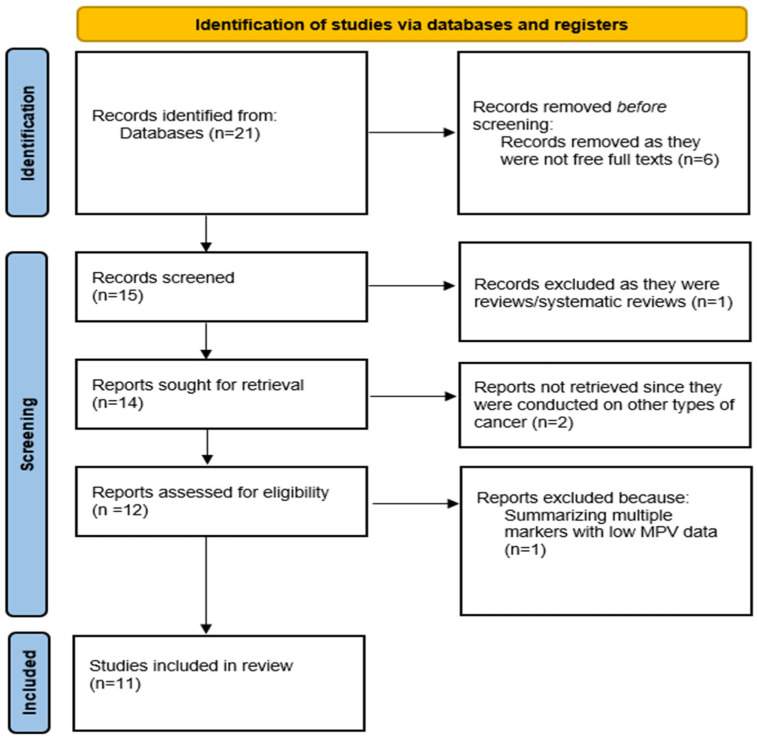
A PRISMA diagram of the current scoping review.

A total of 21 articles were displayed in the PubMed database, but only 15 manuscripts (71.4%) had free full text available, while 6 articles (28.6%) were excluded solely because of the lack of full-text availability. We focused on observational studies (case–control, cohort, cross-sectional) that included adult patients with thyroid nodules or confirmed thyroid cancer, comparing them with healthy controls or patients with benign thyroid disorders. From these 15 manuscripts, we removed the records that were reviews or systematic reviews (1 article) and the articles where the role of MPV was studied in other types of cancer (2 articles). We also removed a manuscript summarizing multiple markers that had low MPV data. Finally, from an initial 21 articles, only 11 met the criteria and were included in the review, as shown below in Figure 1. Of these, all the articles were on human subjects. No formal exclusion based on study quality was applied, consistent with the scoping nature of the review. We highlight that the exclusion of non-open-access studies was based on accessibility issues rather than scientific relevance and may therefore have affected the representativeness of the included evidence.

From these manuscripts, data was extracted and labeled as one or more of the following: MPV values in cancer vs. benign/control groups, diagnostic performance of MPV, associations with clinicopathological features (tumor size, stage, lymph node metastasis), prognostic outcomes (recurrence, survival), or other factors influencing MPV (thyroid function, inflammation, autoimmune disease).

A narrative synthesis was performed because of the heterogeneity and different outcome measures reported in the studies. The results were grouped under thematic categories: MPV in thyroid cancer subtypes, diagnostic value of MPV, prognostic relevance of MPC, and factors influencing MPV interpretation. No statistical pooling or meta-analysis was conducted due to variability in study design, measurement methods, and reporting. Given the absence of a formal risk-of-bias assessment, methodological weaknesses across studies were qualitatively evaluated and explicitly discussed in the Results and Discussion sections. The review followed the main principles of PRISMA-ScR guidelines for transparency in the literature identification and selection. The review protocol was not prospectively registered.

## 3. Results

### 3.1. MPV and Thyroid Malignancies

The characteristics and key findings of the 11 included studies are summarized in Table 1, including study design, MPV values, histological subtypes, and reported diagnostic or prognostic performance, to facilitate critical comparison across the studies.

#### 3.1.1. MPV in Papillary Thyroid Carcinoma

PTC is the most frequent histopathological form of thyroid malignancy and has been the main focus of MPV research. Many studies highlight that MPV is elevated in PTC patients compared with benign nodular goiter or healthy controls, suggesting that malignant transformation is connected to platelet activation. Baldane et al. (2015) showed higher MPV values in PTC patients [~8.05 fL] compared to benign goiter [~7.57 fL] and healthy controls [~7.36 fL], values that decreased after thyroidectomy, supporting the hypothesis that platelet activation is stimulated by the tumor microenvironment [13]. Dincel et al. (2017) and Kutluturk et al. (2019) reported elevations of MPV in PTC cohorts [15,17]. However, Yu et al. (2017) and Martin et al. (2021) documented lower or inconsistent MPV levels in PTC, probably because of differences in population characteristics, methodology, or pre-analytical variability [2,16]. Although most data suggest a higher MPV in PTC, the diagnostic power of MPV alone is limited. Overall, MPV differences in PTC are inconsistent and small, limiting their stand-alone diagnostic relevance.

#### 3.1.2. MPV in Follicular and Other Subtypes

Across the 11 included studies, most of the patients had papillary thyroid carcinoma. A few isolated cases of follicular thyroid carcinoma were reported, being pooled with PTC without separate analysis. Medullary and anaplastic thyroid carcinomas were either absent or represented by single cases, with no meaningful subtype-specific evaluation. No included study evaluated dedicated MPV analyses for FTC, MTC, or ATC.

#### 3.1.3. MPV in Thyroid Microcarcinoma

Papillary thyroid microcarcinoma (PTMC) is a PTC measuring <1 cm. Han et al. (2024) highlighted that MPV combined with red cell distribution width (RDW) improved diagnostic differences in both PTC and PTMC, with MPV alone having a modest predictive capacity [20]. These findings may suggest that high MPV values may be an early event in papillary thyroid carcinogenesis, even in microcarcinomas. However, these observations are based on limited data and should be interpreted cautiously.

### 3.2. Diagnostic Role of MPV

#### 3.2.1. MPV vs. Benign Thyroid Nodules

In most studies, MPV is slightly higher in PTC than in benign nodules or healthy patients. In the study by Baldane et al., they had significantly higher values of MPV preoperative with lower values of MPV after thyroidectomy was performed, indicating a platelet-activation phenomenon linked to the tumor [13]. In other studies with the same comparison batches, the results had relatively small differences (between 0.3 and 0.7 fL). Some studies found no difference or even lower MPV values in cancer groups, highlighting that MPV alone does not reliably distinguish malignant from benignant nodules. However, the trend toward higher MPV values in the malignant groups is frequent and recurrent enough to consider its inclusion as an adjunct marker in the diagnostic process [15,17,18]. The differences observed across studies explain why MPV alone is not a clinically reliable diagnostic tool.

#### 3.2.2. MPV in Combination with Other Hematologic Indices

Considering the fact that MPV values alone are not adequate, several studies have evaluated the impact of MPV by including the marker in a larger hematological panel. Usually, they paired MPV with other inflammatory ratios, such as neutrophil-to-lymphocyte ratio (NLR) and platelet-to-lymphocyte ratio (PLR), showing promising results versus any single marker [20]. For example, studies that combined MPV with NLR reported higher AUCs and better results than for MPV alone, giving insights into associations with nodal disease or recurrence risk. Surprisingly, MPV’s contribution varied across data points, whereas NLR tended to show more consistent links with stage, tumor size, or recurrence. These data suggest that the use of MPV is best when pairing it with markers from a broader inflammatory profile [2,17,18,20]. The contribution of MPV was generally smaller than that of other inflammatory markers.

#### 3.2.3. MPV Integrated with Imaging Systems [TI-RADS]

To enhance preoperative decision-making, researchers tried to integrate hematologic markers with ultrasound-based risk systems such as TI-RADS. Aydin et al. [21] showed that higher MPV and NLR values line up with higher TI-RADS categories, giving small but measurable gains in malignancy prediction compared with TI-RADS alone. However, combining hematological panels with TI-RADS is not enough; fine-needle aspiration (FNA) remains an indispensable tool in the diagnostic procedure [17,20,21]. Even if the addition of MPV and NLR had statistically significant AUC increases compared with TI-RADS alone, the improvements were modest and unlikely to alter clinical management decisions, establishing the role of MPV more as an adjunct than a replacement for fine-needle aspiration.

### 3.3. Prognostic Role of MPV

Prognostic outcomes were categorized as short-term endpoints (e.g., lymph node metastasis) and long-term endpoints (e.g., recurrence or survival).

#### 3.3.1. Association with Lymph Node Metastasis

Some of the studies have examined the relationship between MPV and nodal disease in PTC. Many of them found weak correlations between MPV and extent nodal involvement. However, Li et al. [19] reported an AUC of 0.639 for predicting lymph node metastasis, indicating only modest discrimination below commonly accepted thresholds for clinical actionability [19]. The strength of the association between MPV and lymph node metastasis is modest, although there is some evidence that higher MPV values are linked with more extensive nodal disease.

#### 3.3.2. Association with Recurrence and Survival

The role of MPV in recurrence and survival outcomes is not well established. Li et al. [19] showed AUC values around 0.60 for recurrence prediction, reflecting limited prognostic accuracy, suggesting that MPV provides weak long-term risk stratification when used alone. NLR and the lymphocyte-to-monocyte ratio [LMR] demonstrated stronger prognostic ability than MPV, so evidence directly connecting MPV with overall survival in thyroid cancer patients is limited [19]. In other malignancies [e.g., lung, colorectal], MPV seems to be sometimes associated with survival outcomes [22].

#### 3.3.3. Limitations in Prognostic Utility

Using MPV as a prognostic marker is limited by many factors. Thyroid hormone status, systemic inflammation, or autoimmune thyroid disease can easily influence MPV. Almost all data focus on PTC, with little to no information about the other histotypes (follicular, medullary, or anaplastic carcinoma). Several studies are retrospective and single-centered. The differences in MPV values between the recurrence vs. non-recurrence groups are marginal. MPV can reflect some aspects of tumor aggressiveness, but its prognostic value in thyroid cancer is not established yet, with stronger hematologic markers (e.g., NLR, LMR) being more reliable predictors [19].

### 3.4. Factors Influencing MPV

Thyroid gland status may alter platelet activity. Hyperthyroidism or hypothyroidism can affect MPV whether malignancy is present or not. Measuring TSH, free T4, and free T3 is essential in stratifying patients to avoid false results. Another factor is autoimmune thyroid disease. Some studies have demonstrated elevated MPV values in autoimmune thyroiditis compared with control groups, especially when considering that autoimmune disease frequently coexists with thyroid nodules. Special attention should be paid to pre-analytical and methodological factors, such as the choice of anticoagulant, the time interval between venipuncture and analysis, and the hematology analyzer model; all of these can affect MPV values. So, methodological rigor is mandatory in sample handling for valid MPV values. Besides thyroid-related factors, several medications and comorbidities can influence MPV values. Antiplatelet agents, anticoagulants, diabetes, cardiovascular disease, and obesity are known to affect platelet activation and size. Most included studies did not systematically control for these variables, representing an additional source of potential confounding.

## 4. Discussion

In tumor biology, platelets are active factors contributing to angiogenesis, immune evasion, or metastatic dissemination. Higher MPV values (more metabolically active platelets) can release large amounts of cytokines or growth factors that influence tumor progression. Regarding thyroid cancer, higher MPV is biologically plausible in the platelet–tumor relationship. Most available studies suggest higher MPV values in patients with PTC, supporting the hypothesis that platelet activation may accompany malignant transformation. This biological association is not observed across all studies, and may reflect heterogeneity in patient populations, tumor biology, and thyroid functional status. The substantial heterogeneity observed across studies could be influenced by access-based selection bias. Studies that have restricted access may differ in study design and population size, and their exclusion could have shaped the overall findings. This consideration further supports a cautious interpretation of MPV as a biomarker in thyroid pathology. While MPV can reflect underlying inflammatory and platelet-related mechanisms in thyroid cancer, it should not be interpreted as a tumor-specific signal, but as a non-specific marker of systemic activation.

From a clinical perspective, the utility of MPV is limited. Even though MPV is cheap, routinely available, and easily obtained from blood counts, the diagnostic and prognostic performance in thyroid cancer is modest. Differences in MPV between malignant and benign thyroid nodules are generally small, and AUC values are frequently below thresholds considered clinically acceptable. As demonstrated in the included studies, MPV alone lacks sufficient discriminatory power to guide clinical decision-making. If MPV is paired with multiparametric models, other hematological indices (such as NLR or PLR), imaging systems (TI-RADS), or cytological findings, MPV can provide valuable information. However, its impact is modest and unlikely to replace standard diagnostic tools. MPV should be considered as and adjunctive biomarker rather than a stand-alone diagnostic or prognostic marker in clinical practice.

Regarding other malignancies (lung, colorectal, gastric, or breast cancer), higher MPV values have been linked to tumor presence, stage, or prognosis [22]. Across cancer types, MPV appears to be a general marker of systemic inflammation and platelet activation rather than a disease-specific marker. Similarly to thyroid cancer, associations in other malignancies are inconsistent with limited clinical applicability.

An important matter across both thyroid and non-thyroid cancer literature is the potential for publication bias. Studies reporting positive associations between MPV and malignancy are more likely to be published, particularly in studies where small, retrospective analyses predominate.

### Limitations of the Present Review

A major limitation of this review is the restriction of the literature to studies with free full-text access. Even if this approach enabled detailed data extraction, it represents a non-neutral selection criteria and may have introduced access-based selection bias. Approximately one third of the potentially relevant records identified were excluded because full-text access was not available, independent of study quality or scientific relevance. We acknowledge that this restriction may contribute to the heterogeneity of the results and may have influenced the apparent inconsistency and limited clinical utility of MPV across the studies. We advise that the findings of this review should be interpreted with caution, as the inclusion of non-open-access studies could modify both the strength and direction of the conclusions.

## 5. Future Perspectives

Future studies should focus on large, prospective, multicenter studies. These studies should follow standardized protocols regarding pre-analytical and methodological factors, as well as stratification by thyroid function and autoimmune disease of the patients. Research on the role of MPV in follicular, medullary, and anaplastic carcinomas where evidence is lacking is recommended.

Integrating MPV into larger hematological panels alongside TI-RADS, Bethesda cytology, and molecular panels should also be considered. Additionally, dynamic evaluation of MPV (pre- and postoperative changes) may provide information about recurrence and survival in longitudinal cohorts.

## 6. Conclusions

MPV is a cheap, routinely available biomarker that can reflect platelet activation in thyroid cancer, although with limited disease specificity. In PTCs, MPV is often elevated compared to benign nodules or healthy patients, and MPV values can decrease after thyroidectomy. However, evidence of its prognostic role remains inconsistent.

The current evidence base is largely restricted to PTC, and extrapolation of these findings to other thyroid cancer subtypes, such as follicular, medullary, and anaplastic carcinomas, is not yet justified.

Therefore, MPV should be regarded as an adjunctive marker, with a potential value when combined with other hematological indices and imaging data, not as a stand-alone diagnostic or prognostic test. Before integrating MPV into routine thyroid cancer management, further research is needed with more standardized protocols. This review highlights MPV as an example of a biologically plausible but clinically weak biomarker, illustrating broader challenges in inflammatory biomarker research in thyroid cancer. Altogether, the current evidence suggests that MPV is biologically plausible, but its clinical utility remains limited and inconsistent in thyroid cancer pathology. This conclusion should be viewed in the light of methodological constraints, particularly access-based study selection. Comprehensive future reviews and well-designed prospective studies are required to clarify the true clinical value of MPV.

## Figures and Tables

**Table 1:** Summary of the 11 included studies.

First Author (Year)	Country	Study Design	Sample Size	Histological Type	Thyroid Function/AITD Reported	MPV (Malignant vs. Benign/Control)	Cut-off Value	Outcome Assessed	Diagnostic Performance
Bayhan (2016) [12]	Turkey	Case–control	NR	PTC	NR	Higher in PTC	NR	Diagnosis	NR
Baldane (2015) [13]	Turkey	Case–control	NR	PTC	NR	8.05 vs. 7.57/7.36 fL	NR	Diagnosis	NR
Yaylaci (2016) [14]	Turkey	Case–control	NR	PTC	NR	No significant difference	NR	Diagnosis	NR
Dincel (2017) [15]	Turkey	Case–control	NR	PTC	NR	Higher in PTC	NR	Diagnosis	NR
Yu (2017) [16]	China	Case–control	NR	PTC	NR	Lower/inconsistent	NR	Diagnosis	NR
Kutluturk (2019) [17]	Turkey	Case–control	NR	PTC	NR	Higher in PTC	NR	Diagnosis	NR
Lee (2019) [18]	Korea	Case–control	NR	PTC	NR	Slightly higher in PTC	NR	Diagnosis	NR
Martin (2021) [2]	Romania	Observational	NR	PTC		Inconsistent	NR	Diagnosis	NR
Li (2022) [19]	China	Retrospective cohort	NR	PTC	NR	NR	NR	LNM/recurrence	AUC 0.639 (LNM) 0.60 (recurrence)
Han (2024) [20]	China	Retrospective	NR	PTC/PTMC	NR	Higher MPV and RDW	NR	Diagnosis	Improved AUC vs. MPV alone
Aydin (2025) [21]	Turkey	Retrospective	NR	PTC	NR	Higher MPV with higher TI-RADS	NR	Diagnosis	Modest AUC increase vs. TI-RADS

## Data Availability

No new data were created or analyzed in this study.

## Abbreviations

The following abbreviations are used in this manuscript:

MPV	Mean Platelet Volume
PLT	Platelets
PTC	Papillary Thyroid Carcinoma
NLR	Neutrophil-to-Lymphocyte Ratio
PLR	Platelet-to-Lymphocyte Ratio
